# Exploiting Nanobodies in the Detection and Quantification of Human Growth Hormone *via* Phage-Sandwich Enzyme-Linked Immunosorbent Assay

**DOI:** 10.3389/fendo.2017.00115

**Published:** 2017-05-30

**Authors:** Hossam Murad, Jana Mir Assaad, Rasha Al-Shemali, Abdul Qader Abbady

**Affiliations:** ^1^Department of Molecular Biology and Biotechnology, AECS, Damascus, Syria

**Keywords:** growth hormone, nanobody, camel, phage display, doping detection, recombinant antibody, VHH, biotinylation

## Abstract

**Background:**

Monitoring blood levels of human growth hormone (hGH) in most children with short stature deficiencies is crucial for taking a decision of treatment with extended course of daily and expensive doses of recombinant hGH (rhGH or Somatropin^®^). Besides, misusing of rhGH by sportsmen is banned by the World Anti-Doping Agency and thus sensitive GH-detecting methods are highly welcome in this field. Nanobodies are the tiniest antigen-binding entity derived from camel heavy chain antibodies. They were successfully generated against numerous antigens including hormones.

**Methods:**

A fully nanobody-based sandwich ELISA method was developed in this work for direct measurement of GH in biological samples.

**Results:**

Two major characteristics of nanobody were exploited for this goal: the robust and stable structure of the nanobody (NbGH04) used to capture hGH from tested samples, and the great ability of tailoring, enabling the display of the anti-GH detector nanobody (NbGH07) on the tip of M13-phage. Such huge, stable, and easy-to-prepare phage-Nb was used in ELISA to provide an amplified signal. Previously, NbGH04 was retrieved on immobilized hGH by phage display from a wide “immune” cDNA library prepared from a hGH-immunized camel. Here, and in order to assure epitope heterogeneity, NbGH07 was isolated from the same library using NbGH04-captured hGH as bait. Interaction of both nanobodies with hGH was characterized and compared with different anti-GH nanobodies and antibodies. The sensitivity (~0.5 ng/ml) and stability of the nanobody-base sandwich ELISA were assessed using rhGH before testing in the quantification of hGH in blood sera and cell culture supernatants.

**Conclusion:**

In regard to all advantages of nanobodies; stability, solubility, production affordability in *Escherichia coli*, and gene tailoring, nanobody-based phage sandwich ELISA developed here would provide a valuable method for hGH detection and quantification.

## Introduction

Human growth hormone (hGH), a single-chain polypeptide hormone of 22 kDa and 191 amino acid residues, is synthesized mainly by the acidophilic somatotrophs of the anterior pituitary gland (1). GH is produced and extracted from animals (somatotropin) or synthetized by the recombinant technology (somatropin or rhGH) (2). Medical hGH is given to patients whose pituitary glands produce insufficient quantities of the hormone for normal development and growth (3). Beside its permitted medical application, rhGH is broadly abused by many sportsmen for its lipolitic and anabolic properties. Therefore, it is on the list of substances issued by World Anti-Doping Agency (WADA) as banned for competitive sports. However until recently, a standard test was lacking to detect administrated rhGH (4) in spite of the many proposed assays for measuring GH levels in the blood of young patients (5) or abusing athletes (6). For *in vitro* bioassays, GH measurement depends on its proliferative effect on cultured cell lines which display its specific receptor (7, 8) or through measuring the biological changes of hGH protein markers in the serum (9). Because of their affordability, clinical laboratories are still considering immunoassays for GH measurement in biological samples (10). For years, immunoassays depend on specific anti-GH antibodies (11), recombinant antibody fragments (12), or even DNA aptamers (13).

Alternative means to produce antibodies are recently open through the advances in the field of antibody engineering technology. The recombinant protein constructed from the joined variable parts of conventional antibody, also called the single-chain Fv antibody (scFv), is one of the most successful engineered antibodies with several advantages over the full-length antibody, including the low cost and mass production by fermentation in *Escherichia coli* (14). More importantly, the capacity of tailoring such single chain in order to make fusions with other moieties such as proteins or toxins, resulting in the formation of bi- or multi-functional molecules represent a great advantage of antibody engineering technology (15). Camelids have exceptionally a unique type of antibodies called the heavy chain antibodies (HCAbs), which are naturally devoid of light chains without affecting their capacity of antigen binding (16). Therefore, the recombinant variable domain of HCAb, referred to as nanobody or VHH, is a monomeric structure with astonishing physicochemical characteristics, such as solubility and stability, and a high production yields in *E. coli* or yeast (17). With all their features, nanobodies could overpass the intact antibodies for biotechnological or research purposes and medical applications (18–21) and they might be an efficient alternative to scFv (22, 23). Nanobodies were successfully generated against numerous antigens including various molecules or venoms and even intact pathogens (20, 24–29) as well as purified recombinant proteins (24, 30, 31). Nanobodies are a novel class of affinity binders with promising applications in many fields, such as therapeutics, diagnostics, proteomics, etc. (19).

Previously, we reported the production and characterization of several anti-rhGH nanobodies for use in the field of GH production and detection (32). These nanobodies were retrieved from a large “immune” cDNA library that was prepared from an immunized camel with rhGH fused to the superfolder green fluorescent protein (sfGFP). The current work described our attempt to exploit these nanobodies to develop a full nanobody-based sandwich ELISA for dosing GH concentrations in biological samples. In our seek for better diagnostics and cheaper tools for developing GH assays, such direct method for accurate measuring of GH has a particular importance.

## Materials and Methods

### Antigens and Antibodies

For ELISA and immunoblotting tests, detection of M13 helper phage and M13-Nb was achieved using specific polyclonal antibody (33) and monoclonal antibody anti-M13 conjugated to horseradish peroxidase (HRP, GE Healthcare Life Sciences). For ELISA, detection of antigen-bound nanobodies was mostly accomplished using rabbit anti-6 × His antibody (Bethyl Laboratories Inc.) or with streptavidin–POD (Roche Life Science) when biotinylated nanobodies were used. Subsequent detection of rabbit or mouse antisera was completed using anti-rabbit or anti-mouse antibodies conjugated to HRP for ELISA tests or to alkaline phosphatase for immunoblotting (Bethyl Laboratories Inc.). For nanobody preparation, pMES4 phagemid and *E. coli* strains (TG1 and WK6) were kindly provided by Prof. S. Muyldermans (VUB, Brussels, Belgium). Plasmid constructs for expressing different GH antigens (TEV-GH, GFP-GH, and GFP) were prepared as previously described (32, 34). Expression and purification of these antigens was performed in *E. coli* BL-21(DE3) Gold using standard protocol (32). Commercial un-tagged rhGH was obtained from sigma. d-Biotinoyl-ε-aminocaproic acid-*N*-hydroxysuccinimide ester (Biotin-7-NHS, Roche Life Science) was used to prepare biotinylated nanobodies *via* chemical bioconjugation according to the manufacturer’s instructions.

### Applying Phage Display for Biopanning of GH-Specific Nanobodies

Nanobodies from *E. coli* TG1 library were displayed on the phage particles after infection (~20 times excess phages versus cells) with the M13K07 helper phage (GE Life Sciences). A representative aliquot (1 ml) of the library was grown to mid-logarithmic phase before adding the helper phage. After an overnight growth (in 250 ml), virions were precipitated from the culture supernatant using polyethylene glycol (PEG)/NaCl buffer and then resuspended in a total volume of 1 ml PBS. Bio-panning was performed using MaxiSorp immunotubes (Nunc) precoated with GH or NbGH04 (1 µg/ml). Before adding phages, immunotubes coated with nanobody NbGH04 were washed, blocked in 5% skimmed milk in TBS-T, and then incubated with GH (1 µg/ml) for 1 h at room temperature before adding phage particles (5 × 10^11^ in each immunotubes). GH-specific phages were enriched by several consecutive rounds of *in vitro* selection. From each round and after extensive washing, bound phages were eluted from the immunotubes by incubation for 10 min in triethylamine (100 mM, pH 11.0, Sigma), and eluted particles were immediately neutralized with 1 M Tris–HCl (pH 9.0) and then used to infect exponentially growing *E. coli* TG1 cells. Enrichment of antigen-specific phage-Nb particles was assessed by comparing the number of phages eluted from antigen-coated and -uncoated immunotubes. Individual colonies were picked, and expression of soluble periplasmic nanobody was performed by the addition of 1 mM IPTG. Then, soluble nanobodies from the periplasmic extract were tested in ELISA for their capacity to recognize directly coated or NbGH04-captured GH.

### Expression and Purification of Soluble Nanobodies

Plasmid construct of pMES4 containing NbGH07 nanobody was prepared by a miniprep kit (Qiagen) from *E. coli* TG1, sequenced, and transformed into *E. coli* WK6. Similarly, WK6 of other anti-GH nanobodies were previously prepared to express soluble proteins tagged with C-terminal 6 × His for purification using nickel-charged columns (32). The bacteria were grown in 250 ml shake flasks containing terrific broth medium (1.2% tryptone, 2.4% yeast extracts, 0.8% glycerol, 17 mM KH_2_PO_4_, 72 mM K_2_HPO_4_ with 0.1% glucose and 100 µg/ml ampicillin) to achieve large-scale production of nanobodies. Expression induction with IPTG (1 mM) was performed on the culture after an optical density (at 600 nm) of 0.6–0.9 was reached and further incubation for 16 h at 28°C (35). Periplasmic proteins, including nanobodies, were extracted from pelleted cells by osmotic shock and run through 5 ml nickel charged column on chromatography. After washing, bound nanobodies were recovered with elution buffer containing imidazole (500 mM). Eluted nanobodies were concentrated on Vivaspin tubes with a molecular mass cutoff of 5–10 kDa. Nanobodies concentrations were calculated after measuring the absorption at 280 nm and using the extinction coefficient, as calculated from the amino acid sequence of each nanobody, and were finally adjusted to 1 mg/ml before storage at −20°C. Biconjugation of purified nanobodies (1 mg) with biotin (Biotin-7-NHS) was accomplished according to the manufacturer’s instructions.

### Preparation and Quantification of Phage-Nb Particles

A single colony of *E. coli* TG1 cell containing pMES4-Nb plasmid was inoculated from a Petri dish into 100 ml 2 × TY (1.6% tryptone, 1% yeast extracts and 0.5% NaCl) containing ampicillin (100 µg/ml) and glucose (2%) and in 1-L flask and incubated at 37°C, 200 rpm, till an optical density of OD_600_ = 0.5 was reached. M13K07 helper phages ~10^10^ pfu Ø (>1/20 infection ratio) then were added to the culture, which was incubated for 30 min without shaking at RT, and then for further 30 min with gentle shaking (200 rpm) at 37°C, in order to allow phage infection. After that, cells were pelleted by centrifugation and the medium was replaced by a fresh 2 × TY containing ampicillin (100 µg/ml) and kanamycin (70 µg/ml, Sigma), and the culture was grown overnight at 37°C with shaking (250 rpm). The next day, cells were pelleted by centrifugation, and M13 phages were recovered from the supernatant by precipitation in 5:1 (v:v) volume of PEG (Carl Roth)/NaCl (20% PEG6000 and 2.5 M NaCl). Tubes were mixed gently and incubated for at least 1 h on ice to allow phages precipitation. Finally, phages were recovered by centrifugation, removing supernatant and resuspending the pellet in 0.5-ml PBS containing 7% dimethyl sulfoxide (DMSO, Sigma). Phage concentration was measured either by spectrophotometer at OD_260_ (1 OD = 10^11^ pfu/ml) or by phage sandwich ELISA using rabbit (captor) and mouse (detector) anti-M13 antibodies in the presence of serial concentrations (5 × 10^6^–5 × 10^8^ Ø/ml) of standard M13 helper phage for comparison, as previously described (33).

### Preparing Constructs for Cell Transfection

For this purpose, two plasmids were used, pRSET-a (Invitrogen) and pRSET-TEV-rhGH plasmids (34), to extract the fragments (218 and 786 bp, respectively) corresponding to DNA fragments of pRSET and GH gene with 6 × His tag at the N-terminal in both. Besides, pRSET-TEV-rhGH was modified by the insertion of a linker (GAGAACCTATACTTCCAGGGC) encoding the sequence (ENLYFQ_G), which represents the recognition site for tobacco etch virus (TEV) protease. Both fragments were amplified by PCR using plasmid-specific primers pRSET-to-pcDNA-F (ATAGGCGCGCCTGTACATCATCATCATCATCATGG) and T_7_R (TAGTTATTGCTCAGCGGTGG). In parallel, DNA fragments (~400 bp) of three nanobodies were amplified by PCR from their respective pMES4-Nb plasmids; NbGH01, NbGH07, and NbGFP04 as control (36), using specific primers pMES-to-pcDNA-F (TATGGCGCGCCTGTACAGCTGCAGGAGTCTGGGGGAGGATCGGT) and pMES-to-pcDNA-R (TATGGATCCGCTAGCTCCGGAGGAGACGGTGACCTGAGTCC). PCRs were performed using KOD high-fidelity DNA polymerase (Novagen) to minimize nucleotides mistakes. The amplified fragments were inserted, downstream the secretory (Sec) leader signal (MGWSLILLFLVAVATGVHS) *via Bss*HII/*Eco*RI (for pRSET plasmids) or *Bss*HII/*Bam*HI (for pMES4 plasmids) digestions, into a derivative of pcDNA3 vector (Invitrogen) secreting the anti-FcRI scFv 9E1 (kindly provided by Dr. Oscar Burrone, ICGEB, Trieste, Italy) (37). The final constructs, pcDNA-pRSET, pcDNA-TEV-rhGH, and pcDNA-Nbs, were confirmed by PCR and sequencing before preparation by a transfection-grade Plasmid Midi Prep kit (Qiagen).

### Cell Culture and Transfection

HEK293 cells were grown in Dulbecco’s Modified Eagle Medium supplemented with 10% fetal calf serum and 100 U/ml of penicillin and 100 mg/ml of streptomycin (all from Sigma Chemical). Transient transfections were performed in six-well plates (~5 × 10^5^ cells/well) by standard calcium phosphate technique (38) using 2.5 µg of each plasmid followed by adding serum-free medium 18 h after transfection. For nanobody site-specific biotinylation, a co-transfection with pSECBirA plasmid (1 µg, kindly provided by Dr. Oscar Burrone, ICGEB, Trieste, Italy) (37) was performed, then 18 h after transfection, medium was replaced by serum-free medium supplemented with biotin (0.1 mM, Carl Roth) and incubated for at least 8 h at 37°C. Forty-eight hours post-transfection, cell supernatant was recovered and cells were lysed with lysis buffer (100 µl, 100 mM Tris–HCl, pH 8.0, 250 mM NaCl, 0.5% Nonidet P-40) supplemented with protease inhibitors cocktail (Roche Life Science) and 1 mM phenylmethylsulfonyl fluoride (PMSF, Sigma). A total of 5 µl cell extracts or 20 µl of the supernatants were separated on 15% SDS-PAGE and transferred to nitrocellulose membrane (GE Healthcare) for immunodetection with mouse anti-6 × His tag antibody (R&D systems). GH as well as biotinylated nanobodies in cell supernatants from the different conditions of transfection were tested by ELISA.

### Stability Tests and Enzyme-Linked Immunosorbant Assay (ELISA)

For stability tests, 100 µl of diluted nanobodies (1:10 v:v), R-a-GH antibody (1:50), and phage-Nb particles (10^11^ Ø/ml), in 0.2 µl PCR transparent microtube, were incubated at the indicated conditions (temperature/times) using thermocycler machine or cross-linked under UV^254nm^ lamp to achieve the exposure of the indicated doses (joules). Phage-Nb particles from different conditions were then used to infect exponentially growing *E. coli* TG1 cells, which were then streaked (10 µl) on LB agar supplemented with ampicillin.

For direct ELISA, different antigens were used to coat the wells of Maxisorb 96-well plates (Nunc); rhGH (Somatropin, Sigma), TEV-GH, GFP-GH, or GFP by overnight incubation (at 4°C) with 100 μl/well, prepared at 1 µg/ml in carbonate buffer. Residual protein-binding sites in the wells were blocked for 1 h at 37°C with 5% skimmed milk in TBS-T (20 mM Tris–HCl, 150 mM NaCl, 0.05% Tween-20, pH 7.5). For sandwich ELISA, plates were coated with nanobodies or antibodies (1 µg/ml) in carbonate buffer and after blocking, different antigens, GH derivatives (as indicated), M13 phages (as indicated), blood sera (50% v:v), or cells supernatant (50% v:v), were all prepared in the blocking buffer (1%) and then added to the wells and incubated for further 1 h at room temperature. Dilutions of primary antibodies were prepared and added to the wells according to what was indicated for each experiment. After a number of washes with TBS-T, finale detection was done with either anti-rabbit HRP or with streptavidin–POD in case of biotinylated nanobodies or with mouse anti-M13-HRP antibody in case of phages. Finally, peroxidase substrate 3,3′,5,5′-tetramethylbenzidine (TMB; Sigma) was added and the absorption was measured (at 450 nm) after adding the stopping buffer (1 M of H_2_SO_4_) to neutralize the enzymatic activity of the peroxidase.

### Statistical Analysis

Paired *T*-test was used to compare ELISA signal values of the different conditions of antibodies, nanobodies, and phage-Nb with their respective controls. *P* values lower than 0.01 were considered as statistically significant and marked with * on the different graphs. The calculations were performed using Microsoft Excel.

## Results

### GH Detection Using Nanobody-Based Sandwich ELISA

**Figure 1:**
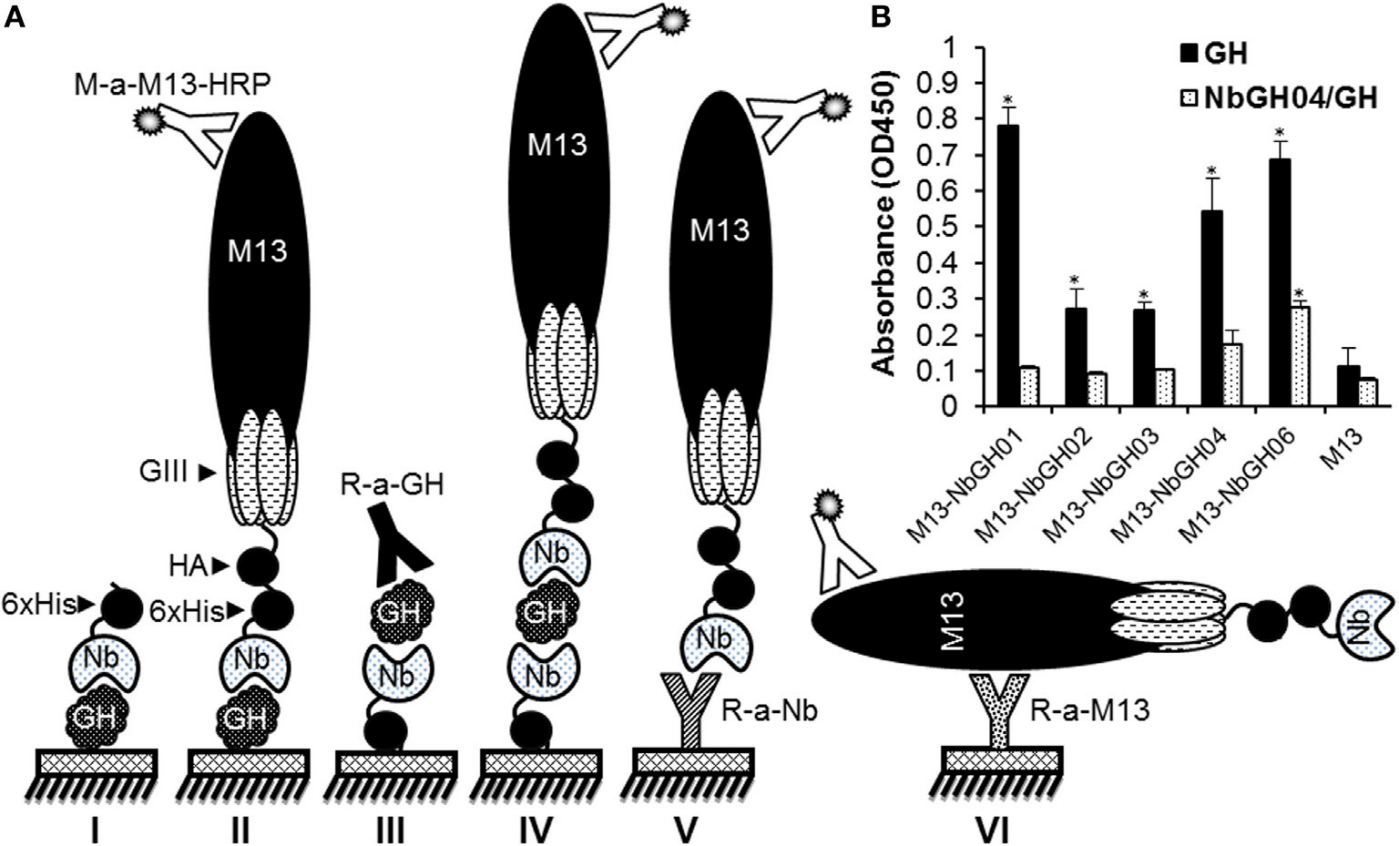
**Different enzyme-linked immunosorbant assay (ELISA) strategies for GH detection using specific nanobodies**. **(A)** Schematic representation of the different methods used for detecting immobilized GH by free (I) or phage-Nb (II). Sandwich ELISA was designed for detecting GH (III and IV), phage-Nb (V), and phages (VI). Detection of phages was always performed using anti-M13-HRP antibody. **(B)** Detection of the immobilized (1 µg/ml) or NbGH04-captured GH (0.2 µg/ml) using five different anti-GH phage-Nb (10^10^ phages/ml). Significant values (*P < 0.01) comparing with their respective controls were marked with * on this graph and the following ones.

In our previously published work, several anti-GH nanobodies, NbGH01, 02, 03, 04, and 06, were retrieved by phage display from a cDNA “immune” library prepared from a camel after immunization with the recombinant GH. Using such nanobodies, different ELISA formats could be conceived for the detection and quantitation of GH (Figure 1A). Generally, all these nanobodies were able to detect immobilized GH in a direct ELISA format, whither they are in a free 6 × His-tagged form (model I) or exposed at the surface of M13 phage (model II). Direct ELISA format requires the immobilization of pure antigens making it useless when impure or mixed antigens needs to be detected, like those in natural samples or body fluids. Interestingly, only NbGH04 and NbGH06 showed a remarkable capacity to capture GH from an impure mixture (bacteria total extract) before being detected by a secondary anti-GH antibody, like a rabbit anti-GH antibody, providing the base to develop a sandwich ELISA format for GH detection (model III). One major characteristic of sandwich ELISA is that the detector antibody should be distinguishable from the capture antibody, especially when a secondary enzyme-conjugated antibody is required for the finale revelation. Nanobody-displaying phages, or phage-Nb particles, are good candidates to act as detector antibodies and the revelation signal could be amplified by using anti-M13-HRP antibody (model IV). However, our different anti-GH nanobodies in their phage-Nb forms have failed to detect efficiently the NbGH04-captured GH (model IV) despite being able to detect a directly immobilized rhGH (model II) (Figure 1B). Besides GH detection, two other sandwich ELISA were developed to detect phage-Nb particles and M13 helper phages using homemade rabbit anti-Nb (model V) and anti-M13 (model VI) as capture antibodies, respectively (Figure 1A).

### Retrieving New Nanobodies against NbGH04-Captured rhGH

**Figure 2:**
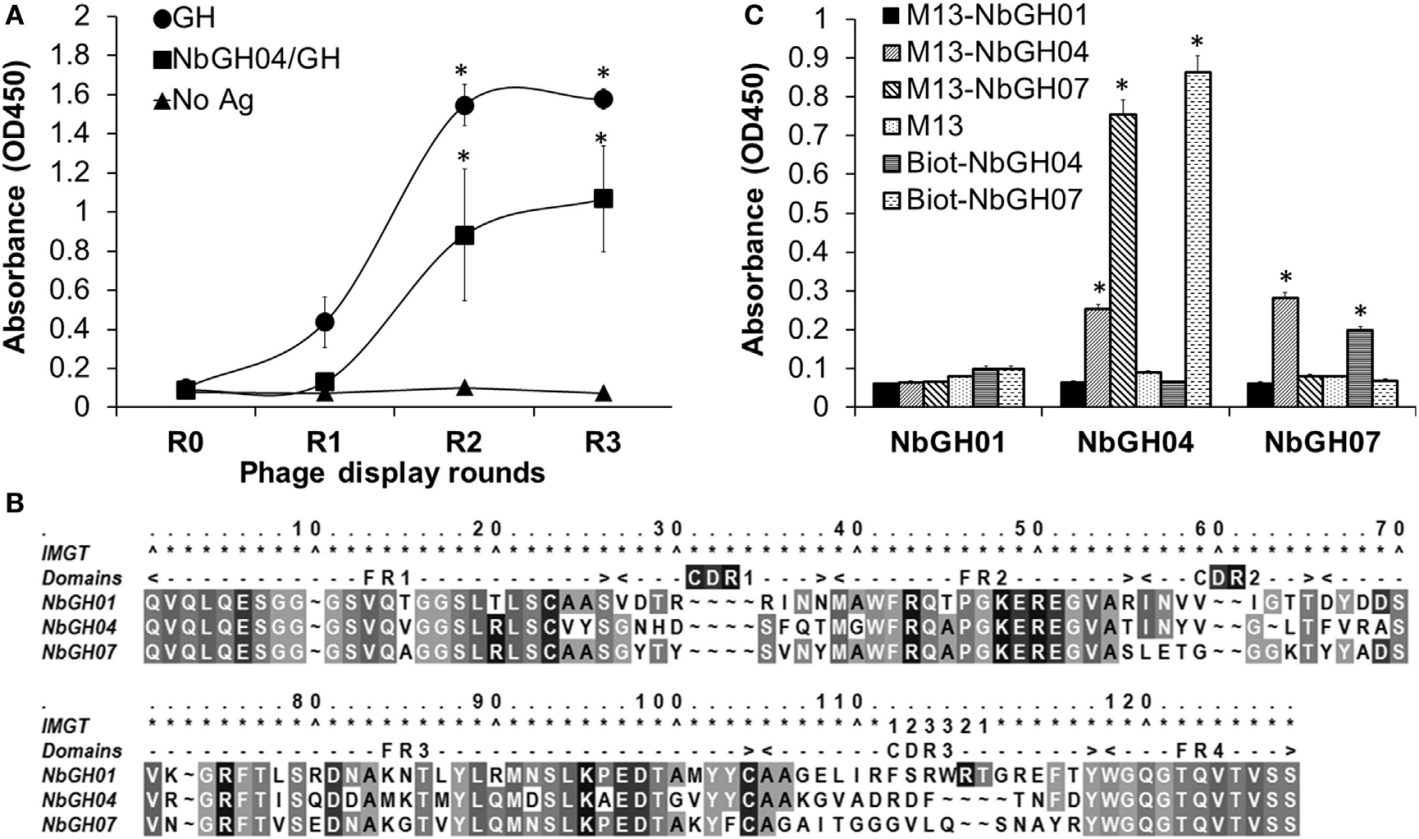
**Phage display panning against GH captured by NbGH04**. **(A)** Phage enzyme-linked immunosorbant assay (ELISA) of the M13 phagemids (10^10^ phages/ml) from three rounds of phage display panning on GH captured by NbGH04. Specificity enrichment was tested against directly immobilized (1 µg/ml) or NbGH04-captured GH (0.2 µg/ml). **(B)** Alignment of the predicted amino acid sequence of nanobodies, where frameworks (FR) and hyper variable regions (CDR) are indicated. Numbers are according to IMGT numbering (39). **(C)** Sandwich ELISA using immobilized nanobodies (0.2 µg/ml), NbGH01, 04, and 07, for capturing GH (0.2 µg/ml) before being detected by phage-Nb (10^10^ phages/ml) or biotinylated nanobodies (0.2 µg/ml).

The failure of phage-Nb particles of the different anti-GH nanobodies to detect NbGH04-captured GH could be explained by an epitope clash since initial phage display bio-panning was performed against an immobilized rhGH. Therefore, isolated nanobodies could be specific to similar or overlapping epitopes of GH. In our previous work, we showed that NbGH04 and NbGH06 could target the same epitope on GH, and NbGH02 and NbGH03 could also be weak competitors for this epitope (32). An exception was NbGH01 which seemed to target a totally different epitope, but at the same time failed in recognizing GH when it is captured by NbGH04 or NbGH06 (data not shown). Therefore, we started a new phage display panning of three rounds on the same anti-GH phage library, but this time using NbGH04-captured rhGH as a bait. Interestingly, a clear enrichment of specific anti-GH phage-Nb particles could be observed from the second round onward, and they were reactive either to the directly immobilized (model II) or to the NbGH04-captured (model IV) rhGH, as shown by a phage ELISA (Figure 2A).

By analyzing the new retrieved anti-GH phage-Nb particles, one nanobody variant, termed NbGH07, with high occurrence in the panning products was identified. After DNA sequencing and amino acid prediction, the alignment of NbGH01, NbGH04, and NbGH07 confirmed its new identity as a VHH with the characteristic amino acid substitutions in the second and third frameworks, with one disulfide bridge (between Cys23 and Cys104) and a relatively long CDR3 (Figure 2B). Sequence comparison between the different anti-GH nanobodies provided evidence that NbGH07 has a distinct sequence, and perhaps B-cell origin, from the previous anti-GH nanobodies. To further confirm the capacity of NbGH07 to detect or capture rhGH in a sandwich ELISA format (model IV), it was prepared as a free and pure recombinant protein from WK6 cells and used to coat an ELISA plate together with NbGH01 and NbGH04 pure nanobodies. Then, captured rhGH was detected using phage-Nb particles of these three nanobodies, and M13 helper phage was used as negative control (Figure 2C). For confirmation, pure NbGH04 and NbGH07 were chemically conjugated to biotin in order to be recognized as detector molecules of rhGH using the system biotin/streptavidin–HRP for their final detection. As expected, NbGH01 was a bad example for capturing rhGH, while NbGH04 was the best especially when the following detection of GH was performed using either phage-Nb or biotinylated forms of NbGH07. The opposite system using NbGH07 for GH capture and phage- or biotinylated-NbGH04 for detection resulted in a lower ELISA signal (Figure 2C).

### Characterizing of NbGH07-Displaying Phages

**Figure 3:**
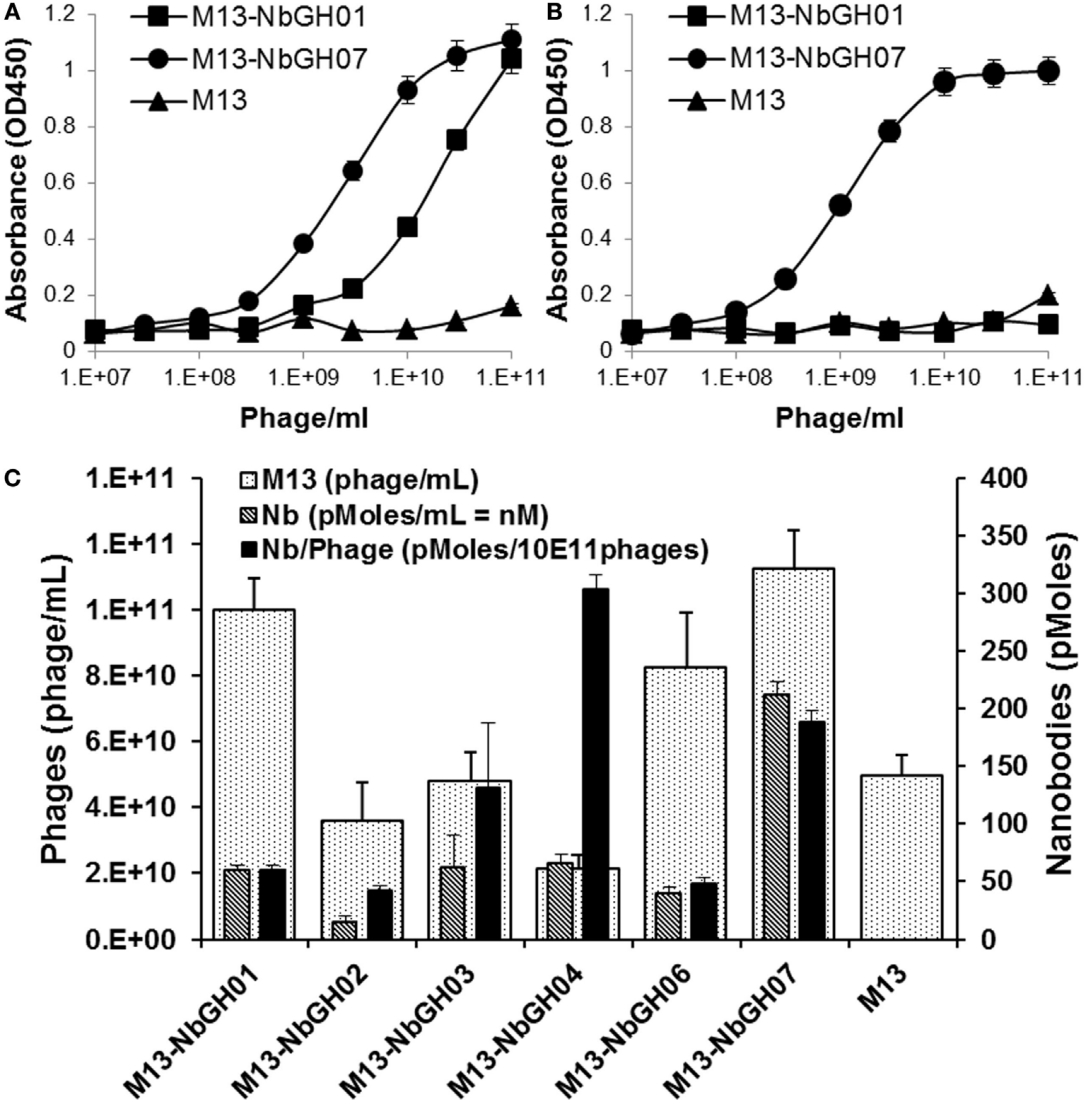
**Characterizing the phages displaying NbGH07**. Phage enzyme-linked immunosorbant assay (ELISA) was performed using serial decimal concentrations (phage\ml) of phage-Nbs, M13-NbGH01 and M13-NbGH07, against a directly immobilized GH [1 µg/ml **(A)**] or after capturing with NbGH04 [0.2 µg/ml **(B)**]. **(C)** A comparison between the different anti-GH phage-Nb prepared from phagemids-containing TG1 cells. Phage yield (phage/ml culture) was calculated using model VI sandwich ELISA. Nanobody yield (pmoles/ml culture) was calculated using model V sandwich ELISA after considering the molecular weight of each nanobody. Display factor (Nb pmoles/10^11^ phages) was calculated for each phage-Nb.

To investigate if the capacity of NbGH07 in detecting rhGH was not related to its availability on the tip of displaying phages comparing to other nanobodies, we performed several ELISA of the already mentioned models. We started by tittering the phage-Nb particles of NbGH07 against a directly immobilized (Figure 3A) or NbGH04-captured (Figure 3B) rhGH, and similar scale of concentrations of M13-NbGH01 and M13-helper phages was used for comparison. Interestingly, similar curves were observed in both conditions using M13-NbGH07 but not M13-NbGH01. The effective concentration resulting in 50% of the maximal signal (EC^50^) for M13-NbGH07 was estimated of about 3 × 10^9^ and 10^9^ phage/ml for detecting immobilized or captured rhGH, respectively. EC^50^ of M13-NbGH01 for the detection of only immobilized GH was about 3 × 10^10^ phage/ml.

Another interesting question was regarding the production yield of each phage-Nb from the TG1 transformed with the encoding plasmids (pMES4-Nb) of the six anti-GH nanobodies. As determined using standard M13 phage-ELISA (model VI), production yield of phages ranged between 2 × 10^10^ and 1 × 10^11^ phage/ml of bacterial culture, and M13-NbGH07 was among the best produced phages, exceeding the value (5 × 10^10^ phage/ml) of M13 helper phage that was used as control (Figure 3C). Consequently, another sandwich ELISA (model V) was performed on the bacterial supernatant in order to estimate nanobodies availability (picomoles per mL) in the samples of different Nb-producing TG1 cells. Once more, M13-NbGH07 was the best in nanobody content among the tested nanobodies; however, its display ratio (picomoles of nanobodies per 10^11^ phages) was the second after M13-NbGH04 (Figure 3C).

### Specificity and Sensitivity of NbGH07 Phage-Nb Particles

**Figure 4:**
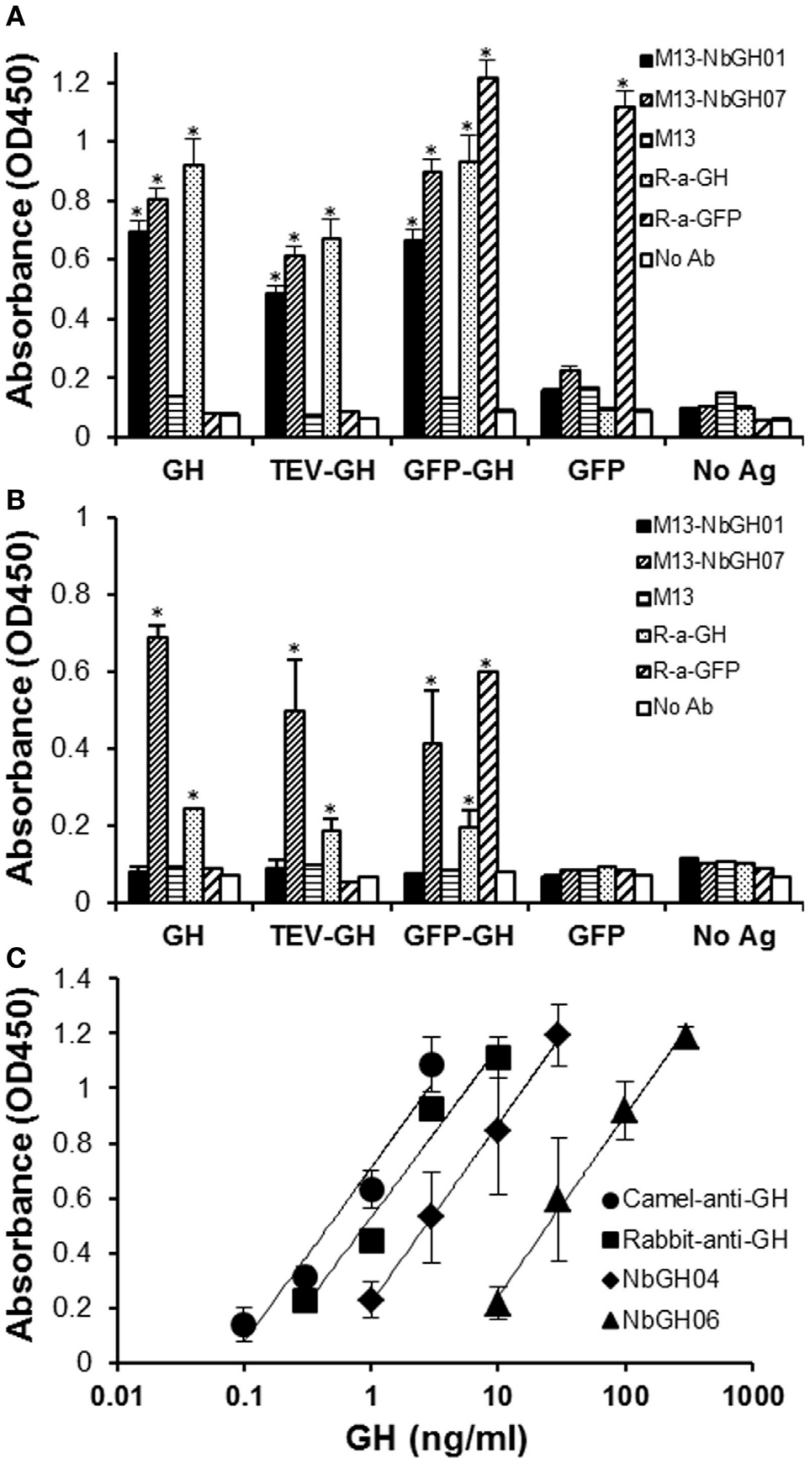
**Evaluation of the specificity and sensitivity of M13-NbGH07 toward GH**. Enzyme-linked immunosorbant assay (ELISA) was performed using phage-Nbs, M13-NbGH01 and M13-NbGH07, and specific anti-GH and anti-GFP antibodies (1:3,000) against a directly immobilized antigens [1 µg/ml **(A)**], GH, TEV-GH, GFP-GH, or GFP, or after a capturing step with NbGH04 [0.2 µg/ml **(B)**]. **(C)** Detection sensitivity of M13-NbGH07 (10^10^ phages/ml) was tested in sandwich ELISA using different capturing agents (0.2 µg/ml); camel-anti-GH, rabbit-anti-GH, NbGH04, and NbGH06, in the presence of serial decimal concentrations of GH (nanograms per milliliter).

Then, we tested the capacity of M13-NbGH07 to recognize different forms of GH; rhGH (or Somatropin), TEV-GH, GH fusion with GFP and GFP as control, either immobilized directly (Figure 4A) or captured using NbGH04 nanobody (Figure 4B). For comparison, different antigens were detected using M13-NbGH01 and M13 helper phage as well as using specific anti-GH and anti-GFP rabbit polyclonal antibodies. As expected, all immobilized GH antigens were detected with the two anti-GH phage-Nbs and antibody, and in the contrary, R-anti-GFP antibody was the only to recognize GFP-GH and GFP. However, only M13-NbGH07 was able to recognize NbGH04-captured GH antigens in the second ELISA and to a lesser extent was the R-anti-GH, while R-anti-GFP recognized the fusion protein GFP-GH, but not the GFP, since it was captured by anti-GH NbGH04 nanobody.

The sensitivity of M13-NbGH07 to low concentrations of rhGH was tested but this time by varying the molecules, NbGH04, NbGH06, rabbit anti-GH, or camel anti-GH, used to capture these concentrations (Figure 4C). Apparently, full antibodies, from rabbit or camel, are more efficient in capturing low concentrations of GH reaching 0.1 ng/ml, while NbGH04 capture a linear range of concentrations from 1 to 30 ng/ml and NbGH06 detect a range 10 times higher.

### Stability of M13-NbGH07

**Figure 5:**
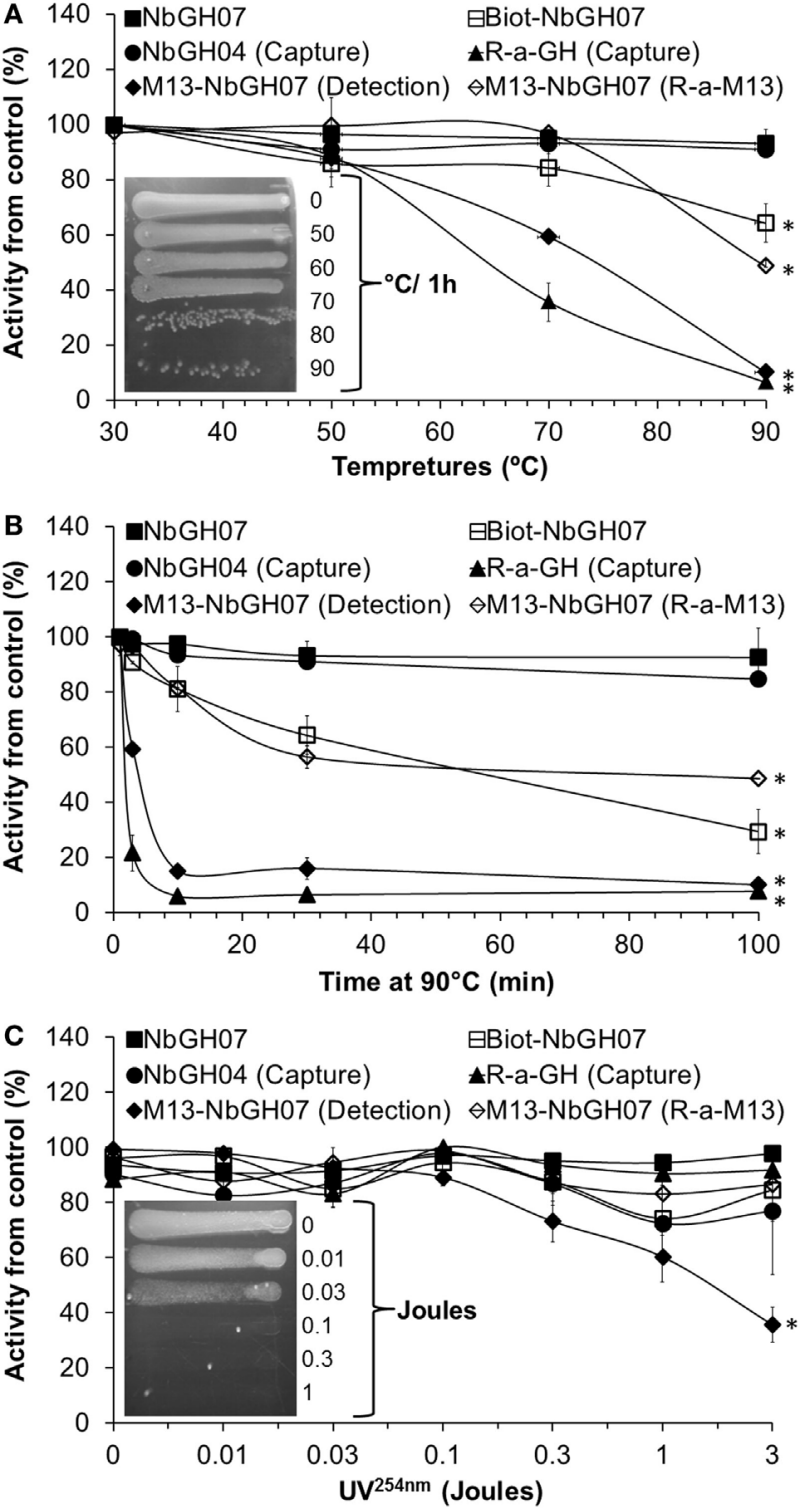
**Heat and UV stability tests of M13-NbGH07**. NbGH07 (free, biotinylated, and phage-Nb), free NbGH04, and rabbit anti-GH were incubated for 0.5 h at different temperatures **(A)**, at 90°C for different times **(B)** or exposed to different doses (joules) of UV^254nm^ **(C)**. Treated samples were tested in sandwich enzyme-linked immunosorbant assay (ELISA) for capturing (Model III) or detecting (Model I) of GH. The integrity of treated phage-Nb of NbGH07 was tested using sandwich ELISA (model VI). Insets in panels **(A,C)** are for LB agar plates (with ampicillin) showing streaks of TG1 cells infected with the phage-Nb particles after exposure to the different temperatures (for 0.5 h) or UV^254nm^ doses.

Nanobody has a stable structure that is resistant to harsh conditions such as extreme pH and temperatures. However, in our conditions, nanobodies are used as one structural entity with M13; thus, it was important to test the stability of phage-Nb particles under different conditions. As expected, NbGH07 and NbGH04 free nanobodies were extremely stable after 30-min exposure to increasing temperatures up to 90°C and retained their capacity to detect (NbGH07) or to capture (NbGH04) rhGH (Figure 5A). Similarly, biotinylated NbGH07 was resistant to temperatures put lost some activity to detect GH at very high degrees, while the polyclonal anti-GH antibody has lost about 50% of activity (to capture GH) at 70°C and was totally futile at 90°C. Concerning M13-NbGH07, the virus function (as detector) and structure, as tested by ELISA model IV and VI respectively, were intact up to 50°C incubation and start to disintegrate at 70°C (with significant 45% decrease in functionality) and lost all its capacity to detect rhGH at 90°C. TG1 infection test showed that M13 infectivity was apparently untouched when heating at 70°C or below and decreased drastically from 80 to 90°C (Figure 5A, inset).

To conceive what really happened at very high temperatures, antibodies were incubated for different times (up to 100 min) at 90°C before being tested in different ELISA formats. As before, free nanobodies retained their full reactivity, and only the biotinylated NbGH07 lost reactivity in function of time. M13-NbGH07 behaved in a similar manner as full antibodies, as they have all lost a great deal of activity since the first minutes of exposure to this temperature and were totally worthless after 100 min (Figure 5B).

Exposure to a range of doses (between 0.01 and 3 J) of UV^254nm^ has less destructive effect on protein structure than did the heat, since most of the tested antibodies (including nanobodies) retained more than 80% of their activity after radiation with 3 J. However, M13 was very sensitive to UV, which resulted in a dose-dependent decrease in phage-Nb activity in the range between 0.1 and 3 J (significant 60% activity loss) (Figure 5C). M13 infectivity after UV exposure was also tested and showed that a dose of 0.1 J was enough to fully destroy the capacity of the bacteriophage to infect TG1 cells (Figure 5C, inset).

### Application of M13-NbGH07 Phage ELISA for GH Quantitation in Biological Samples

**Figure 6:**
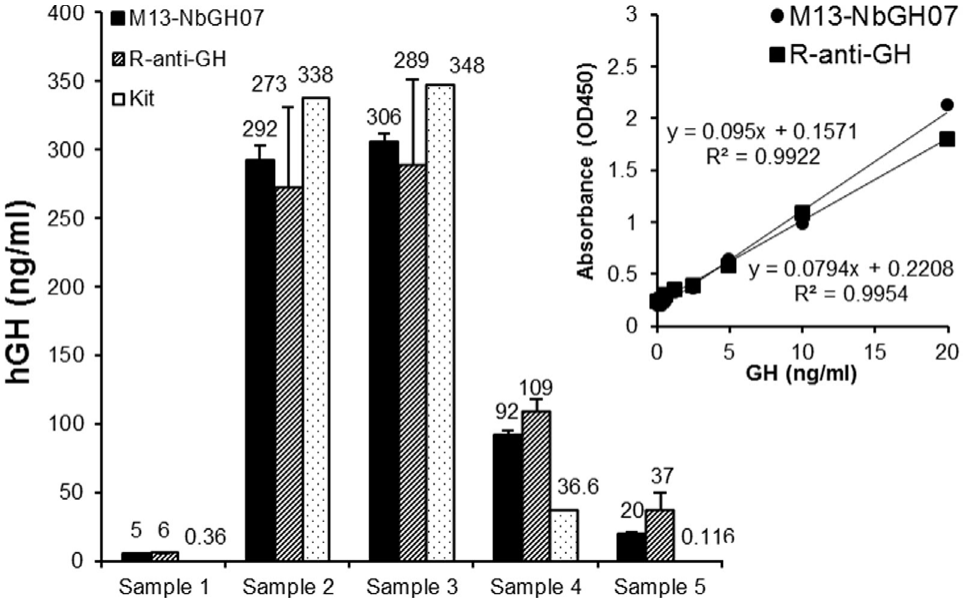
**Quantitation of blood content of GH using M13-NbGH07 phage enzyme-linked immunosorbant assay (ELISA)**. GH content (nanograms per milliliter) was measured in five plasma samples using phage (model IV) or conventional (model III) sandwich ELISA. Absorbance values were calculated using the linear equations of the standard curve of known GH concentrations resulted from the two different ELISA models (inset). GH concentration values in the plasma samples were determined using a commercial ELISA test (Kit).

Sandwich ELISA (models III and IV) were applied on blood sera from five different persons in order to quantitate the exact content of GH in these samples (Figure 6). To give an exact concentration value (nanograms per milliliter), standard curves were established (for the two models of ELISA) using serial concentrations of rhGH, and the linear fit equation was extracted for each curve and subsequently used to calculate the GH concentration in the blood samples (Figure 6, inset). Interestingly, detection of NbGH04-captured GH from blood sera either with M13-NbGH07 (model IV) or with rabbit anti-GH antibody (model III) have resulted in relatively similar values for each of the five samples and with no significant differences from those given by a commercial kit for GH quantitation in blood.

**Figure 7:**
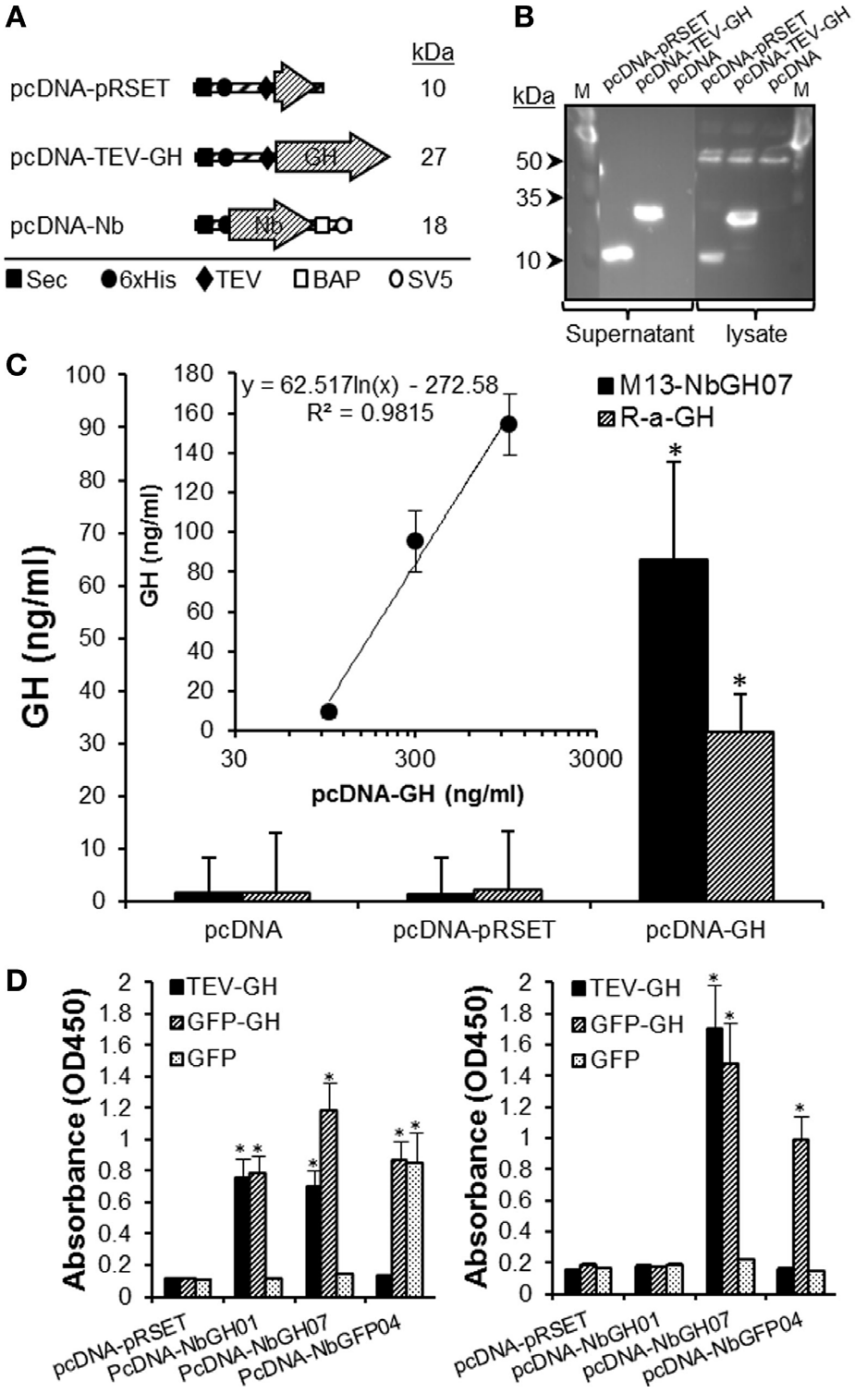
**Quantitation of GH in the supernatant of HEK293 cell using M13-NbGH07 phage enzyme-linked immunosorbant assay (ELISA)**. **(A)** Schematic representation of the recombinant proteins; pRSET, TEV-GH, and nanobodies secreted by HEK293 transfected cells with the corresponding pcDNA plasmids. The theoretical molecular size (kiloDaltons) is shown to the right of each recombinant construct. Positions of the different elements, the Sec signal, 6 × His tag, TEV, BAP, and SV5, are indicated using specific symbols ■, ●, ♦, □, and ○, respectively. **(B)** Detection of pRSET and TEV-GH proteins in the lysate (10 µl) and supernatant (25 µl) of 48 h transfected HEK293 cells after SDS-PAGE (15%) separation by immunoblotting using anti-6 × His antibody. **(C)** Measuring of GH (nanograms per milliliter) in the cell supernatants was performed using phage (model IV) or conventional (model III) sandwich ELISA. (Inset) Quantitation of GH concentration (nanograms per milliliter), using phage ELISA (model III), in the supernatant (25 µl) of HEK293 cells after 48 h of transfection with serial concentrations of pcDNA-GH plasmid. **(D)** Biotinylated nanobodies (NbGH01, NbGH07, and NbGFP04) secreted in the supernatant of transfected cells were tested in conventional (model I where 6 × His tag is replaced by the biotin group, left panel) and sandwich (model IV where M13 is replaced by the biotin group and using NbGH04 as bait, right panel) ELISA. Streptavidin–HRP (1:1,000) was used to reveal the biotinylated detector nanobodies bound to GH, GFP-GH, and GFP antigens.

We evaluated the use of M13-NbGH07 phage ELISA for detecting rhGH secreted in the supernatant of cell cultures. For this aim a special plasmid (pcDNA-TEV-GH) was constructed and used to transiently transfect human embryonic kidney cells HEK293T. This plasmid permits the cell to secret an N-terminal 6 × His-tagged rhGH of 27 kDa (Figure 7A). It was possible to detect this protein in the supernatant and cell lysate 48 h post-transfection by immunoblotting with an anti-6 × His tag antibody (Figure 7B). Levels of rhGH in the supernatant were detected using M13-NbGH07 (model IV) and rabbit anti-GH (model III) sandwich ELISA, after capturing with NbGH04 (Figure 7C). Both techniques were efficient for detecting significantly secreted rhGH from HEK293T cells transfected with pcDNA-TEV-GH plasmid, and not the control plasmid pcDNA-pRSET. Furthermore, a logical correlation, between the amount of plasmid used for cell transfection and the concentration of the secreted rhGH (nanograms per milliliter) in the supernatant, was found by applying any of the two ELISA models (Figure 7C, inset).

Finally, we took advantage of this eukaryotic system to confirm the capacity of NbGH07 to detect immobilized or NbGH04-captured hGH. For this aim, a special pcDNA plasmid was constructed to secret nanobodies with an *in vivo* site-specific biotinylation in the presence of bacterial BirA enzyme (Figure 7A). Interestingly, all three secreted nanobodies (NbGH01, NbGH07, and NbGFP04) were biotinylated (on the BAP motif) and functional as they were able to detect specifically their immobilized antigens (TEV-GH, GFP-GH, and GFP) and were revealed by a streptavidin–HRP conjugate (Figure 7D, left panel). As expected, only NbGH07, and not NbGH01, was able to detect significantly NbGH04-captured rhGH (free or fused to GFP) (Figure 7D, right panel). As control, biotinylated NbGFP04 was able to detect specifically immobilized GFP-GH and GFP and only NbGH04-captured GFP-GH (Figure 7D).

## Discussion

Camel nanobodies gained recently an increasing attention and started to overrun many fields of medicine, agriculture, and even industry and captured the interest of many researchers around the world (40). Nanobodies have proven to be efficient and valuable molecular tools for many applications of biotechnology (41). Their intact, small, and stable structure beside their remarkable specificity make them competent to conventional antibodies (42). Furthermore, nanobodies are produced in laboratory through a genetic procedure, starting from camel immunization, library construction, and then selection of specific binders by a high throughput phage display technology (42). The final outcome of the procedure, which may last for 4 months, is the isolation of several individual *E. coli* colonies; each expresses an antigen-specific nanobody and at the same time harbors the plasmid with the gene fragment encoding this nanobody. The conservation of such valuable nanobodies is simply done by recovering and storing their encoding plasmids. By contrast, in case of full monoclonal antibodies, the conservation of their producing hybridoma cell line risk accidental contamination and requires sophisticated equipment (e.g., minus 80 freezer and liquid nitrogen containers). Comparing to recombinant immune fragment from conventional antibodies (scFv), nanobodies seems to be very interesting alternatives because of their stable structure resembling the original structure of camel VHH. Therefore, a relatively small cDNA library of about 10^6^ variants could be a true representative of camel immune response and thus, is largely enough to retrieve antigen-specific binders (42), whereas a library of at least 10^9^ variants is needed in case of scFv, where VH and VL domains from the immune pool are scrambled then rebound artificially with a joint (14). One big advantage of scFv over nanobody is the host animal, especially that camelids unlike mice, rabbits, or chickens that are used for scFv do not exist naturally in most developed countries and their maintenance is considered as a challenge for laboratories working with recombinant antibody fragments. Fortunately, Syria is a natural habitat for one-humped Arabian camel, giving us a great advantage in this technology and versatile molecular tools (nanobodies) to exploit in different biotechnological and medical applications.

Since their retrieval from an “immune” cDNA library, prepared from an immunized Arabian camel, anti-GH nanobodies were designated for detection purposes (32). In this work, we started by exploiting one important result from a previous work, in which we showed that NbGH04 and NbGH06 were not only reactive to the immobilized or rabbit-IgG-captured rhGH but also were particularly able to capture rhGH. Unfortunately, captured rhGH could only be detected using the polyclonal anti-GH antibody (rabbit IgG) and not any of the other nanobodies, especially that it is not yet clear whether they all detect different epitopes of GH or not. Here, we confirmed that different anti-GH phage-Nb particles, and despite being all able to detect immobilized GH, could not detect NbGH04-captured rhGH, accenting on the need for new anti-GH nanobodies that might can. The availability of a relatively big nanobody library of 5 × 10^8^ transformants, with high percentage (~90%) of correct nanobody–gene inserts, could reflect a considerable diversity of nanobodies, and therefore, the already obtained GH-specific nanobodies should not be considered as the final outcome of this library, but instead many other interesting binders are still need to be retrieved by phage display but using different GH forms or using alternative strategies rather than the direct panning on immobilized rhGH. Therefore, rhGH was first captured using immobilized NbGH04 before incubation with the total phage-Nb particles of the M13-infected library, and indeed it worked, and we obtained a clear enrichment of phage-Nb particles that are able to bind to immobilized or captured rhGH. Analyzing of these phage-Nb particles resulted in the identification of the sixth anti-GH nanobody, called NbGH07. This new nanobody was able to bind to NbGH04-captured rhGH, either when it was exposed on the phage (M13-NbGH07) or free expressed then bioconjugated to biotin (Biot-NbGH07) to be discriminated from the captor nanobody NbGH04. Apparently, the epitopes targeted by NbGH07 and NbGH04 are different; however, reversing sandwich ELISA order, by using NbGH07 as a captor and NbGH04 (in form of Nb-phage or biotinylated) as a detector, resulted in a low ELISA signal. This could be related in some way to the weak capacity of NbGH07 to capture rhGH when it is adsorbed on a plastic surface.

Phage display is a sensitive, efficient, and convenient technology of functional proteomics for the elucidation of protein–protein interactions, disease mechanisms, or therapeutic targets. We tried in this work to examine the possibility to exploit the principle of phage display in developing a sandwich ELISA method for hGH quantification. The versatility of phage display technology has proven to be a method to select specific binders simply by changing the selection methodology. M13 phage has an elevated growth rate, and its amplification in liquid bacteria culture requires only 1–3 h. It was clearly demonstrated that at least 10^13^ phage particles could be generated per 1 L *E. coli* culture of which 10^9^ phages/well are enough for hGH detection in ELISA. In other words, the quantity of phage-Nb particles from 1-L culture, which are simply recovered from the supernatant by precipitation with PEG/NaCl method, is largely sufficient for 10^4^ ELISA tests. The phage-Nb of NbGH07 showed high values not only in term of phage recovery (10^14^) from 1 L culture but also in term of nanobody display ratio on these phages. Structurally, NbGH07 nucleotide sequence was analyzed by DNA sequencing and the prediction of its amino acid composition confirmed its new identity and its differences from the five previous anti-GH nanobodies (32). In addition to the conserved bridge between Cys23 and Cys104, that is present in all the clones, NbGH07 does not show the extra interloop disulfide bond, frequently occurring between CDR1 and CDR3 in camel nanobodies, that was only observed in NbGH02 and 06 (32). However, this extra bond did not seem to be crucial for antigen recognition or binding neither for nanobody stability under extreme conditions of heat or UV since NbGH04 and NbGH07, which both lake the extra disulfide bond, retained their full function after exposure to such conditions.

In phage display, bound phages are measured by ELISA using antibodies specific for the capsid proteins. Although anti-M13 p8 monoclonal antibody conjugated to HRP (from GE healthcare) has been used for phage detection by ELISA, the capturing step was accomplished using an in-house polyclonal anti-M13 antibody. The procedure of phage quantification by sandwich ELISA using these two anti-M13 was previously setup and compared to plaque assay (33). Detection of hGH-bound phage-Nb particles, especially NbGH07, could be achieved using both anti-M13 antibodies; however, the method was faster with the commercial monoclonal antibody but the signal was higher with the home-made one. Structurally, M13 filamentous phage consists of 2,700 copies of capsid p8 and 7 copies of lateral capsid p3 and 4 other capsids (43). Each engineered M13 phage is capable of displaying ~3–5 copies of a nanobody fused to the N-terminus of capsid p3 with the remaining copies of capsid p3 provided by host bacteria. Displaying the nanobodies on the tips of M13 is simply done by fusion of nanobody gene fragments with the p3 coat protein. This step is usually insured during the library preparation when the PCR-amplified DNA fragments of the different nanobodies are cloned in the phagemid pMES4 and just upstream of the *G3 gene*. Later, when the phagemids-transformed bacteria but in contact with helper M13 phage, spontaneously, is starts to bud a modified verions with the encoded nanobodies displayed on their tips. Apparently, the structure of the displayed nanobody can affect the efficiency of phages formation by the transformed bacteria. NbGH07-transformed TG1 *E. coli* has one of the elevated phage-production yield (10^14^ phages/l) and the nanobody displaying ratio of this nanobody in considerably high compared to the other nanobodies.

Combining the phage presentation of nanobodies with the capacity to produce biotinylated particles at site-specific reaction in the presence of BirA enzyme could be of great importance in the field of hGH diagnosis. Furthermore, the anti-hGH nanobody-based sandwich ELISA was also applicable when M13 moiety of phage-NbGH07 was replaced with a biotin tag, as a distinguishing tag for the detector nanobody from the captor one. The designed primers, plasmid constructs, and the optimized method developed here for the production of secreted and biotinylated nanobodies could be easily applied to any available nanobodies. In fact, the system used here to produce *in vivo*-secreted and biotinylated proteins in eukaryotic cells was previously described by Burrone group (44). Biotinylation is an interesting alternative for purification and detection of nanobodies after being bound to their specific antigens using several biotechnological methods. Indeed, the secreted nanobodies from HEK293T transfected cells were biotinylated and their specificity toward their respective antigens was intact. Furthermore, developing a system to produce secreted nanobodies by eukaryotic cells could be of a high importance in studying their physiological role on their native antigens.

GH immunoassays vary significantly because of the differences in calibration, isoform recognition, interference with GH-binding proteins, and antibody specificity (45). There are several isoforms of GH circulating in blood together with the binding proteins (growth hormone-binding protein) making the measurement of GH a complicated task. Cell culture proliferation bioassay, depending on the expressed GH receptor by these cell lines, had recorded the lowest detection limit (~0.5 ng/ml), and had the highest specificity for GH in spite of the non-specific interference by factors present in serum (7, 8). Meanwhile, different immunoassays (RIA, IRMA, and ELISA) are generally used in clinical laboratories because of sensitivity, speed, and accessibility (5). In fact, two conventional ELISA formats, competitive and sandwich ELISA, are frequently applied in hGH quantification when it exists in impure mixture, as in blood samples or tissue culture supernatants. Competitive ELISA is frequently applied with small antigens, especially those composed of a single epitope such as haptens, and require a continuous supply of the pure antigen that plays the role of competitor or a bait for the detector antibody. Sandwich ELISA, described in this work, has one major advantage over competitive ELISA in that it provides double certitude of the measured antigen, since two different epitopes should be at least targeted, one by the captor and the other by the detector antibodies. Interestingly, most isolated nanobodies, including NbGH07, showed high EC_50_, reaching ~1 nM, and acceptable sensitivity toward rhGH in sandwich ELISA, as most of them were able to detect GH at very low concentrations, ranging from 0.5 to 10 ng/ml.

## Conclusion

This study describes for the first time the optimization of a fully nanobody-based sandwich ELISA method for the detection and quantification of GH in the blood or in the supernatant of cultured cells. Distinguishing the GH-detector nanobody from the captor one required the exploitation of one of the most important stages of nanobody production, which is the phage display, where nanobodies make parts of a huge viral structure and retain their full capacity to detect the antigens. Such structure is ideal for ELISA considering the high signal amplification that it may provide using a secondary anti-M13 antibody for final revelation. Considering all interesting characteristics of this Nb-phage sandwich ELISA, it may represent a powerful tool for GH detection and quantification, especially for GH miss-use by sportsmen which is strictly panned by WADA.

## Declarations

### Ethics Approval and Consent to Participate

The experimental and scientific content of this study have been validated and approved by the Institutional Review Board and Ethical Committee of the Atomic Energy Commission of Syria (AECS). The committee’s reference number for the project is 194/2016.

### Consent for Publication

Every one of the five patients was informed about the study, and a written consent was signed either by the patient or his/her parent for blood sample which was used exclusively for GH diagnostic tests.

### Availability of Data and Materials

The data set(s) supporting the results of this article is(are) included within the article [and its additional file(s)].

## Author Contributions

HM and AA conceived and designed the experiments. JA and RA-S performed the experiments. AA analyzed data. HM and AA wrote the manuscript with contributions from all authors. All the authors read and approved the final manuscript.

## Conflict of Interest Statement

The authors declare that the research was conducted in the absence of any commercial or financial relationships that could be construed as a potential conflict of interest.

## Abbreviations

ELISA, enzyme-linked immunosorbent assay; HCAb, heavy chain antibody; HRP, horseradish peroxidase; scFv, single-chain antibody variable fragment; WADA, World Anti-Doping Agency.

## References

[1] Baumann GP. Growth hormone isoforms. Growth Horm IGF Res (2009) 19(4):333–40. 10.1016/j.ghir.2009.04.011 19467614

[2] De Palo EF De Filippis V Gatti R Spinella P. Growth hormone isoforms and segments/fragments: molecular structure and laboratory measurement. Clin Chim Acta (2006) 364(1–2):67–76. 10.1016/j.cca.2005.06.009 16194529

[3] Vance ML Mauras N Growth hormone therapy in adults and children. N Engl J Med (1999) 341(16):1206–16. 10.1056/NEJM199910143411607 10519899

[4] McHugh CM Park RT Sonksen PH Holt RI. Challenges in detecting the abuse of growth hormone in sport. Clin Chem (2005) 51(9):1587–93. 10.1373/clinchem.2005.047845 16020502

[5] Popii V Baumann G. Laboratory measurement of growth hormone. Clin Chim Acta (2004) 350(1–2):1–16. 10.1016/j.cccn.2004.06.007 15530455

[6] He C Wu M. Detection of doping with recombinant human growth hormone. Bioanalysis (2009) 1(5):953–65. 10.4155/bio.09.85 21083065

[7] Ishikawa M Nimura A Horikawa R Katsumata N Arisaka O Wada M A novel specific bioassay for serum human growth hormone. J Clin Endocrinol Metab (2000) 85(11):4274–9. 10.1210/jcem.85.11.6983 11095467

[8] Maimaiti M Tanahashi Y Mohri Z Fujieda K. Development of a bioassay system for human growth hormone determination with close correlation to immunoassay. J Clin Lab Anal (2012) 26(5):328–35. 10.1002/jcla.21527 23001977 PMC6807445

[9] Jing J Zhou X He C Zhang L Yang S Xu Y Biomarker detection of rhGH doping: an excretion study. Drug Test Anal (2012) 4(10):739–44. 10.1002/dta.1423 23074170

[10] Bidlingmaier M Freda PU. Measurement of human growth hormone by immunoassays: current status, unsolved problems and clinical consequences. Growth Horm IGF Res (2010) 20(1):19–25. 10.1016/j.ghir.2009.09.005 19818659 PMC7748084

[11] Thomas A Schanzer W Delahaut P Thevis M. Immunoaffinity purification of peptide hormones prior to liquid chromatography-mass spectrometry in doping controls. Methods (2012) 56(2):230–5. 10.1016/j.ymeth.2011.08.009 21871962

[12] Bird RE Hardman KD Jacobson JW Johnson S Kaufman BM Lee SM Single-chain antigen-binding proteins. Science (1988) 242(4877):423–6. 10.1126/science.3140379 3140379

[13] Bruno JG Carrillo MP Phillips T Edge A. Discrimination of recombinant from natural human growth hormone using DNA aptamers. J Biomol Tech (2011) 22(1):27–36. 21455479 PMC3059541

[14] Ahmad ZA Yeap SK Ali AM Ho WY Alitheen NB Hamid M. scFv antibody: principles and clinical application. Clin Dev Immunol (2012) 2012:980250. 10.1155/2012/980250 22474489 PMC3312285

[15] Liu X Wang H Liang Y Yang J Zhang H Lei H Production and characterization of a single-chain Fv antibody-alkaline phosphatase fusion protein specific for clenbuterol. Mol Biotechnol (2010) 45(1):56–64. 10.1007/s12033-010-9240-2 20087689

[16] De Genst E Saerens D Muyldermans S Conrath K Antibody repertoire development in camelids. Dev Comp Immunol (2006) 30(1–2):187–98. 10.1016/j.dci.2005.06.010 16051357

[17] Dumoulin M Conrath K Van Meirhaeghe A Meersman F Heremans K Frenken LG Single-domain antibody fragments with high conformational stability. Protein Sci (2002) 11(3):500–15. 10.1110/ps.34602 11847273 PMC2373476

[18] De Genst E Silence K Decanniere K Conrath K Loris R Kinne J Molecular basis for the preferential cleft recognition by dromedary heavy-chain antibodies. Proc Natl Acad Sci U S A (2006) 103(12):4586–91. 10.1073/pnas.0505379103 16537393 PMC1450215

[19] Saerens D Ghassabeh GH Muyldermans S. Single-domain antibodies as building blocks for novel therapeutics. Curr Opin Pharmacol (2008) 8(5):600–8. 10.1016/j.coph.2008.07.006 18691671

[20] Deckers N Saerens D Kanobana K Conrath K Victor B Wernery U Nanobodies, a promising tool for species-specific diagnosis of *Taenia solium* cysticercosis. Int J Parasitol (2009) 39(5):625–33. 10.1016/j.ijpara.2008.10.012 19041315

[21] Muyldermans S Baral TN Retamozzo VC De Baetselier P De Genst E Kinne J Camelid immunoglobulins and nanobody technology. Vet Immunol Immunopathol (2009) 128(1–3):178–83. 10.1016/j.vetimm.2008.10.299 19026455

[22] Van Bockstaele F Holz JB Revets H. The development of nanobodies for therapeutic applications. Curr Opin Investig Drugs (2009) 10(11):1212–24. 19876789

[23] Wesolowski J Alzogaray V Reyelt J Unger M Juarez K Urrutia M Single domain antibodies: promising experimental and therapeutic tools in infection and immunity. Med Microbiol Immunol (2009) 198(3):157–74. 10.1007/s00430-009-0116-7 19529959 PMC2714450

[24] Conrath KE Lauwereys M Galleni M Matagne A Frere JM Kinne J Beta-lactamase inhibitors derived from single-domain antibody fragments elicited in the camelidae. Antimicrob Agents Chemother (2001) 45(10):2807–12. 10.1128/AAC.45.10.2807-2812.2001 11557473 PMC90735

[25] Stijlemans B Conrath K Cortez-Retamozo V Van Xong H Wyns L Senter P Efficient targeting of conserved cryptic epitopes of infectious agents by single domain antibodies. African trypanosomes as paradigm. J Biol Chem (2004) 279(2):1256–61. 10.1074/jbc.M307341200 14527957

[26] El Khattabi M Adams H Heezius E Hermans P Detmers F Maassen B Llama single-chain antibody that blocks lipopolysaccharide binding and signaling: prospects for therapeutic applications. Clin Vaccine Immunol (2006) 13(10):1079–86. 10.1128/CVI.00107-06 16928888 PMC1595319

[27] Abderrazek RB Hmila I Vincke C Benlasfar Z Pellis M Dabbek H Identification of potent nanobodies to neutralize the most poisonous polypeptide from scorpion venom. Biochem J (2009) 424(2):263–72. 10.1042/BJ20090697 19732033

[28] Harmsen MM van Solt CB Fijten HP. Enhancement of toxin- and virus-neutralizing capacity of single-domain antibody fragments by N-glycosylation. Appl Microbiol Biotechnol (2009) 84(6):1087–94. 10.1007/s00253-009-2029-1 19455325 PMC2755796

[29] Lam AY Pardon E Korotkov KV Hol WG Steyaert J. Nanobody-aided structure determination of the EpsI: EpsJ pseudopilin heterodimer from *Vibrio vulnificus*. J Struct Biol (2009) 166(1):8–15. 10.1016/j.jsb.2008.11.008 19118632 PMC4107884

[30] Bakhtiari SH Rahbarizadeh F Hasannia S Ahmadvand D Iri-Sofla FJ Rasaee MJ. Anti-MUC1 nanobody can redirect T-body cytotoxic effector function. Hybridoma (Larchmt) (2009) 28(2):85–92. 10.1089/hyb.2008.0079 19249993

[31] Roovers RC Vosjan MJ Laeremans T El Khoulati R de Bruin RC Ferguson KM A biparatopic anti-EGFR nanobody efficiently inhibits solid tumour growth. Int J Cancer (2011) 129(8):2013–24. 10.1002/ijc.26145 21520037 PMC4197845

[32] Abbady AQ Al-Shemali R Mir Assaad J Murad H. Generation and characterization of nanobodies against rhGH expressed as sfGFP fusion protein. Gen Comp Endocrinol (2014) 204:33–42. 10.1016/j.ygcen.2014.05.018 24859761

[33] Twair A Al-Okla S Kawas H Abbady AQ Production of polyclonal antibody against M13 phage for application in nanobody technology. Adv Biol Res (Rennes) (2013) 7(11):3216–23.

[34] Murad H Ali B Makeya R Abbady AQ. Prokaryotic overexpression of TEV-rhGH and characterization of its polyclonal antibody. Gene (2014) 542(1):69–76. 10.1016/j.gene.2014.02.016 24534464

[35] Arbabi Ghahroudi M Desmyter A Wyns L Hamers R Muyldermans S. Selection and identification of single domain antibody fragments from camel heavy-chain antibodies. FEBS Lett (1997) 414(3):521–6. 10.1016/S0014-5793(97)01062-4 9323027

[36] Twair A Al-Okla S Zarkawi M Abbady AQ. Characterization of camel nanobodies specific for superfolder GFP fusion proteins. Mol Biol Rep (2014) 41(10):6887–98. 10.1007/s11033-014-3575-x 25085037

[37] Predonzani A Arnoldi F Lopez-Requena A Burrone OR. In vivo site-specific biotinylation of proteins within the secretory pathway using a single vector system. BMC Biotechnol (2008) 8:41. 10.1186/1472-6750-8-41 18423015 PMC2373293

[38] Sambrook J Fritsch EF Maniatis T Molecular Cloning, a Laboratory Manual. New York, NY: Cold Spring Harbor Laboratory Press (1989).

[39] Lefranc MP Pommie C Ruiz M Giudicelli V Foulquier E Truong L IMGT unique numbering for immunoglobulin and T cell receptor variable domains and Ig superfamily V-like domains. Dev Comp Immunol (2003) 27(1):55–77. 10.1016/S0145-305X(02)00039-3 12477501

[40] Hassanzadeh-Ghassabeh G Devoogdt N De Pauw P Vincke C Muyldermans S Nanobodies and their potential applications. Nanomedicine (Lond) (2013) 8(6):1013–26. 10.2217/nnm.13.86 23730699

[41] De Meyer T Muyldermans S Depicker A. Nanobody-based products as research and diagnostic tools. Trends Biotechnol (2014) 32(5):263–70. 10.1016/j.tibtech.2014.03.001 24698358

[42] Muyldermans S. Nanobodies: natural single-domain antibodies. Annu Rev Biochem (2013) 82:775–97. 10.1146/annurev-biochem-063011-092449 23495938

[43] Levisson M Spruijt RB Winkel IN Kengen SW van der Oost J. Phage display of engineered binding proteins. Methods Mol Biol (2014) 1129:211–29. 10.1007/978-1-62703-977-2_19 24648080

[44] De Lorenzo G Eichwald C Schraner EM Nicolin V Bortul R Mano M Production of in vivo-biotinylated rotavirus particles. J Gen Virol (2012) 93(Pt 7):1474–82. 10.1099/vir.0.040089-0 22442113

[45] Manolopoulou J Alami Y Petersenn S Schopohl J Wu Z Strasburger CJ Automated 22-kD growth hormone-specific assay without interference from pegvisomant. Clin Chem (2012) 58(10):1446–56. 10.1373/clinchem.2012.188128 22908135

